# Supplementary material for: Safety of Co-Administration Versus Separate Administration of the Same Vaccines in Children: A Systematic Literature Review
Source: Vaccines (Basel). 2019 Dec 31;8(1):12. doi: 10.3390/vaccines8010012 (PMC7157665; doi:10.3390/vaccines8010012)
Supplement: Supplementary file 1 [file vaccines-08-00012-s001.zip › PhD_LitRev_List vaccine abbreviations_REVRev Table-1.docx]

**Supplementary table 2.** List of abbreviations.

| **Abbreviation** | **Meaning** |
| --- | --- |
| AEFI | Adverse event following immunisation |
| aRR | Adjusted relative risk |
| DT | Diphtheria and tetanus toxoids vaccine |
| DTaP | Diphtheria and tetanus toxoids and acellular pertussis vaccine |
| DTaP-HepB-IPV/Hib | Diphtheria and tetanus toxoids and acellular pertussis adsorbed, hepatitis B, inactivated poliovirus and *Haemophilus influenzae* type b conjugate vaccine |
| DTaP-IPV | Diphtheria and tetanus toxoids and acellular pertussis adsorbed and inactivated poliovirus vaccine |
| DTaP-IPV/Hib | Diphtheria and tetanus toxoids and acellular pertussis adsorbed, inactivated poliovirus and *Haemophilus influenzae* type b conjugate vaccine |
| HepA | Hepatitis A vaccine |
| HepA-HepB | Hepatitis A inactivated and hepatitis B vaccine |
| HepB | Hepatitis B vaccine |
| HepB-Hib | Hepatitis B and *Haemophilus influenzae* type b conjugate vaccine |
| Hib | *Haemophilus influenzae* type b conjugate vaccine |
| Hib-MenCY | *Haemophilus influenzae* type b conjugate and bivalent meningococcal conjugate vaccine |
| HPV | Human papillomavirus vaccine |
| IIV (H1N1) | Inactivated influenza vaccine |
| IIV3 | Trivalent inactivated influenza vaccine |
| IPV | Inactivated poliovirus vaccine |
| IQR | Interquartile range |
| JE | Japanese encephalitis vaccine |
| LAIV | Live attenuated influenza vaccine |
| LJEV | Live attenuated Japanese encephalitis vaccine |
| MenACWY | Quadrivalent meningococcal conjugate vaccine |
| MenB | Serogroup B meningococcal vaccine |
| MenC | Serogroup C meningococcal vaccine |
| MMR | Measles, mumps, and rubella vaccine |
| MMRV | Measles, mumps, rubella, and varicella vaccine |
| MR | Measles rubella vaccine |
| OPV | Oral polio vaccine |
| PCV7 | Pneumococcal conjugate (7-valent) vaccine |
| PCV13 | Pneumococcal conjugate (13-valent) vaccine |
| RCT | Randomised clinical trial |
| RD | Risk difference |
| RR | Relative risk |
| RV | Rotavirus vaccine |
| Td | Tetanus and diphtheria toxoids vaccine |
| Tdap | Tetanus toxoid, reduced diphtheria toxoid and acellular pertussis vaccine |
| VAR | Varicella vaccine |
| YF | Yellow fever vaccine |
