# Supplementary material for: Safety of Co-Administration Versus Separate Administration of the Same Vaccines in Children: A Systematic Literature Review
Source: Vaccines (Basel). 2019 Dec 31;8(1):12. doi: 10.3390/vaccines8010012 (PMC7157665; doi:10.3390/vaccines8010012)
Supplement: Supplementary file 1 [file vaccines-08-00012-s001.zip › Suppl Table-2-final.docx]

**Supplementary table 1.** Characteristics of studies comparing co-administration versus separate administration.

| **Adverse Events** | **Vaccines co-administered** | **Study type** | **Sample size** | **Age min^+^** | **Age max^+^** | **Country** | **Reference** |
| --- | --- | --- | --- | --- | --- | --- | --- |
| 1. **Statistically significantly more AE^#^** | | | | | | | |
| Inj. site pain, Inj. site bruising | MenACWY + Tdap + HPV | RCT | 1042 | 120.0 | 204.0 | USA | Reisinger, Keith S.; Block, Stan L.; Collins-Ogle, Michelle; Marchant, Colin; Catlett, Melissa; Radley, David; Sings, Heather L.; Haupt, Richard M.; Garner, Elizabeth I. O. (2010) [[1]](https://www.zotero.org/google-docs/?tKySZY) |
| Inj. site swelling | MenACWY + Tdap + 9vHPV | RCT | 1241 | 132.0 | 180.0 | Chile, Columbia, Mexico, Peru, USA | Schilling, Andrea; Parra, Mercedes Macias; Gutierrez, Maricruz; Restrepo, Jaime; Ucros, Santiago; Herrera, Teobaldo; Engel, Eli; Huicho, Luis; Shew, Marcia; Maansson, Roger; Caldwell, Nicole; Luxembourg, Alain; Sobanjo-ter Meulen, Ajoke (2015) [[2]](https://www.zotero.org/google-docs/?GAEdDh) |
| Inj. site tenderness, Headache | Td + MMR +HepB | RCT | 197 | 132.0 | 144.0 | USA | Cassidy, William M.; Jones, Glenn; Williams, Karen; Deforest, Adamadia; Forghani, Bagher; Virella, Gabriel; Venters, Charmaine (2005) [[3]](https://www.zotero.org/google-docs/?LuVKyJ) |
| Fever/Pyrexia | DTaP-HepB-IPV/Hib + PCV7 | RCT | 266 | 1.9 | 3.7 | France, Germany | Olivier; Belohradsky; Stojanov; Bonnet; Petersen; Liese (2008) [[4]](https://www.zotero.org/google-docs/?4CWAbM) |
| Fever/Pyrexia | PCV13 + IIV3 | Prospective observational cohort | 530 | 6.0 | 23.0 | USA | Stockwell, Melissa S.; Broder, Karen; LaRussa, Philip; Lewis, Paige; Fernandez, Nadira; Sharma, Devindra; Barrett, Angela; Sosa, Jose; Vellozzi, Claudia (2014) [[5]](https://www.zotero.org/google-docs/?D2dPau) |
| Vomiting | DTaP-IPV/Hib + MenC + RV5 | RCT | 247 | 1.4 | 1.6 | Finland | Vesikari, Timo; Karvonen, Aino; Borrow, Ray; Kitchin, Nick; Baudin, Martine; Thomas, Stephane; Fiquet, Anne (2011) [[6]](https://www.zotero.org/google-docs/?ilL3xX) |
| Myalgia | MenACWY + Tdap + HPV | RCT (phase 3) | 1283 | 132.0 | 216.0 | USA | Wheeler, Cosette M.; Harvey, Bryan M.; Pichichero, Michael E.; Simon, Michael W.; Combs, Stephen P.; Blatter, Mark M.; Marshall, Gary S.; Catteau, Gregory; Dobbelaere, Kurt; Descamps, Dominique; Dubin, Gary; Schuind, Anne (2011) [[7]](https://www.zotero.org/google-docs/?5MywnB) |
| Overall | DTaP-IPV/Hib + MMR | RCT | 176 | 12.0 | 12.0 | Israel | Shneyer, Elena; Strulov, Avshalom; Rosenfeld, Yaakov (2009) [[8]](https://www.zotero.org/google-docs/?HomFrK) |
| 1. **Statistically significantly less AE^#^** | | | | | | | |
| Inj. site erythema | DTaP-HepB-IPV/Hib + MenC | RCT | 452 | 1.9 | 7.0 | Spain | Tejedor, Juan C.; Omenaca, Félix; Garcia-Sicilia, José; Verdaguer, Joaquim; Van Esso, Diego; Esporrin, Carlos; Molina, Vicente; Muro, Marta; Mares, Josep; Enrubia, Manuel; Moraga, Fernando; Garcia-Corbeira, Pilar; Dobbelaere, Kurt; Schuerman, Lode (2004) [[9]](https://www.zotero.org/google-docs/?6nBOKk) |
| Conjunctivitis | OPV + LAIV | RCT | 2503 | 6.0 | 35.0 | Bangladesh, Chile, Columbia, Malaysia, Peru, Philippines, Thailand | Breiman, Robert F.; Brooks, W. Abdullah; Goswami, Doli; Lagos, Rosanna; Borja-Tabora, Charissa; Lanata, Claudio F.; Londono, Jaime A. Cespedes; Lum, Lucy Chai See; Rappaport, Ruth; Razmpour, Ahmad; Walker, Robert E.; Gruber, William C.; Forrest, Bruce D. (2009) [[10]](https://www.zotero.org/google-docs/?gIoiIO) |
| Rash, Rhinorrhoea | MMR + VAR + Hib-HepB | RCT | 822 | 12.0 | 15.0 | USA | Hesley, Teresa M.; Reisinger, Keith S.; Sullivan, Bradley J.; Jensen, Erin H.; Stasiorowski, Susan; Meechan, Cathy; Chan, Christina Y.; West, David J. (2004) [[11]](https://www.zotero.org/google-docs/?mNpdYy) |
| Insomnia, Nasopharyngitis | PCV7 + MMRV | RCT | 1027 | 12.0 | 15.0 | USA | Leonardi, Michael; Bromberg, Kenneth; Baxter, Roger; Gardner, Julie L.; Klopfer, Stephanie; Nicholson, Ouzama; Brockley, Michael; Trammel, James; Leamy, Vicky; Williams, Wendy; Kuter, Barbara; Schodel, Florian (2011) [[12]](https://www.zotero.org/google-docs/?tWr6pV) |
| Diarrhoea, Fever/Pyrexia | DTaP-IPV + RV5 | RCT | 192 | 1.4 | 2.6 | Japan | Tanaka, Yoshiyuki; Yokokawa, Ruriko; Rong, Han Shi; Kishino, Hiroyuki; Stek, Jon E.; Nelson, Margaret; Lawrence, Jody (2017) [[13]](https://www.zotero.org/google-docs/?XwvqQR) |
| 1. **No statistically significantly differences in AE^#^** | | | | | | | |
|  | MenACWY + Tdap + HPV | RCT (phase 3) | 1620 | 132.0 | 216.0 | Costa Rica | Arguedas, A.; Soley, C.; Loaiza, C.; Rincon, G.; Guevara, S.; Perez, A.; Porras, W.; Alvarado, O.; Aguilar, L.; Abdelnour, A.; Grunwald, U.; Bedell, L.; Anemona, A.; Dull, P. M. (2010) [[14]](https://www.zotero.org/google-docs/?3xrKnV) |
|  | OPV + RV + routine vaccines | RCT | 735 | 1.4 | 2.8 | Brazil, Costa Rica, Guatemala, Mexico | Ciarlet, Max; Sani-Grosso, Ramei; Yuan, Guojun; Liu, Guanghan F.; Heaton, Penny M.; Gottesdiener, Keith M.; Arredondo, Jose L.; Schodel, Florian (2008) [[15]](https://www.zotero.org/google-docs/?3qdGu9) |
|  | IPV + YF | RCT (phase 4) | 1504 | 9.0 | 10.0 | The Gambia | Clarke, Ed; Saidu, Yauba; Adetifa, Jan U.; Adigweme, Ikechukwu; Hydara, Mariama Badjie; Bashorun, Adedapo O; Moneke-Anyanwoke, Ngozi; Umesi, Ama; Roberts, Elishia; Cham, Pa Modou; Okoye, Michael E.; Brown, Kevin E.; Niedrig, Matthias; Chowdhury, Panchali Roy; Clemens, Ralf; Bandyopadhyay, Ananda S.; Mueller, Jenny; Jeffries, David J.; Kampmann, Beate (2016) [[16]](https://www.zotero.org/google-docs/?UyCgPm) |
|  | MR + IPV |  |  |  |  |  |  |
|  | MR + IPV + YF |  |  |  |  |  |  |
|  | MR + YF |  |  |  |  |  |  |
|  | MenACWY-TT + PCV13 | RCT (phase 3) | 802 | 12.0 | 14.0 | Australia, Canada, Czech Republic, Panama, South Africa, Turkey | Cutland, Clare L.; Nolan, Terry; Halperin, Scott A.; Kurugol, Zafer; Ahmed, Khatija; Perrett, Kirsten P.; Richmond, Peter; Marshall, Helen S.; Ceyhan, Mehmet; Kolhe, Devayani; Hezareh, Marjan; Van Der Wielen, Marie (2018) [[17]](https://www.zotero.org/google-docs/?QsBA3x) |
|  | DTaP-HepB-IPV/Hib + MMRV | RCT | 963 | 12.0 | 23.0 | Germany, Italy | Deichmann, Klaus A.; Ferrera, Giuseppe; Tran, Clement; Thomas, Stephane; Eymin, Cecile; Baudin, Martine (2015) [[18]](https://www.zotero.org/google-docs/?C8Rrx8) |
|  | MMRV + MenC | RCT (phase 3b) | 716 | 13.0 | 15.0 | Italy | Durando, Paolo; Esposito, Susanna; Bona, Gianni; Cuccia, Mario; Desole, Maria Giuseppina; Ferrera, Giuseppe; Gabutti, Giovanni; Pellegrino, Angelo; Salvini, Filippo; Henry, Ouzama; Povey, Michael; Marchetti, Federico (2016) [[19]](https://www.zotero.org/google-docs/?hdRQr9) |
|  | IIV3 + IIV (H1N1) | RCT | 64 | 120.0 | 252.0 | Italy | Esposito, Susanna; Meregalli, Elisa; Daleno, Cristina; Ghio, Luciana; Tagliabue, Claudia; Valzano, Antonia; Serra, Domenico; Galeone, Carlotta; Edefonti, Alberto; Principi, Nicola (2011) [[20]](https://www.zotero.org/google-docs/?7E069p) |
|  | IIV3 + IIV (H1N1) | RCT | 72 | 108.0 | 240.0 | Italy | Esposito, Susanna; Tagliaferri, Laura; Daleno, Cristina; Valzano, Antonia; Picciolli, Irene; Tel, Francesca; Prunotto, Giulia; Serra, Domenico; Galeone, Carlotta; Plebani, Anna; Principi, Nicola (2011) [[21]](https://www.zotero.org/google-docs/?ZsJyMn) |
|  | MenACWY + Tdap | RCT (phase 3) | 1072 | 132.0 | 300.0 | Italy | Gasparini, Roberto; Conversano, Michele; Bona, Gianni; Gabutti, Giovanni; Anemona, Alessandra; Dull, Peter M.; Ceddia, Francesca (2010) [[22]](https://www.zotero.org/google-docs/?GcQj4C) |
|  | MMR + VAR | RCT | 299 | 12.0 | 24.0 | Philippines | Gatchalian, Salvacion; Leboulleux, Didier; Desauziers, Eric; Bermal, Nancy; Borja-Tabora, Charissa (2003) [[23]](https://www.zotero.org/google-docs/?BLrCaR) |
|  | MMR + VAR | RCT | 300 | 12.0 | 24.0 | Philippines | Gatchalian, Salvacion; Tabora, Charissa; Bermal, Nancy; Leboulleux, Didier; Desauziers, Eric (2004) [[24]](https://www.zotero.org/google-docs/?fEIr4O) |
|  | Measles + LJEV | RCT | 600 | 8.0 | 10.0 | Philippines | Gatchalian, Salvacion; Yao, Yafu; Zhou, Benli; Zhang, Lei; Yoksan, Sutee; Kelly, Kim; Neuzil, Kathleen M.; Yaich, Mansour; Jacobson, Julie (2008) [[25]](https://www.zotero.org/google-docs/?RXqoyg) |
|  | DTaP-HepB-IPV/Hib + PCV7 | RCT (phase 2b) | 460 | 2.0 | 15.0 | Canada | Halperin, Scott A.; Tapiero, Bruce; Dionne, Marc; Meekison, William; Diaz-Mitoma, Francisco; Zickler, Paul; Rubin, Earl; Embree, Joanne; Bhuyan, Prakash; Lee, Andrew; Li, Minran; Tomovici, Antigona (2014) [[26]](https://www.zotero.org/google-docs/?id0Chi) |
|  | MMR + JE-CV | RCT | 550 | 12.0 | 18.0 | Taiwan | Huang, Li-Min; Lin, Tzou-Yien; Chiu, Cheng-Hsun; Chiu, Nan-Chang; Chen, Po-Yen; Yeh, Shu-Jen; Boaz, Mark; Hutagalung, Yanee; Bouckenooghe, Alain; Feroldi, Emmanuel (2014) [[27]](https://www.zotero.org/google-docs/?ojmDMP) |
|  | MenACWY + Td | Surveillance data | 128,297* | 108.0 | 300.0 | USA | Jackson, Lisa A.; Yu, Onchee; Nelson, Jennifer; Belongia, Edward A.; Hambidge, Simon J.; Baxter, Roger; Naleway, Allison; Nordin, James; Baggs, James; Iskander, John (2009) [[28]](https://www.zotero.org/google-docs/?RD0rQI) |
|  | MenACWY + Tdap |  |  |  |  |  |  |
|  | DTaP-IPV/Hib or DTaP-HepB-IPV/Hib + MMR or MMRV | Case control | 590 | 16.0 | 23.0 | Canada | Kiely, Marilou; Billard, Marie-Noëlle; Toth, Eveline; Zafack, Joseline G.; Landry, Monique; Skowronski, Danuta M.; De Serres, Gaston (2018) [[29]](https://www.zotero.org/google-docs/?C1sv56) |
|  | MenACWY + DTaP-HepB-IPV/Hib | RCT (phase 3) | 793 | 12.0 | 23.0 | Austria, Germany, Greece | Knuf, Markus; Pantazi-Chatzikonstantinou, Anna; Pfletschinger, Ulrich; Tichmann-Schumann, Irmingard; Maurer, Hartwig; Maurer, Lothar; Fischbach, Thomas; Zinke, Henrike; Pankow-Culot, Heidemarie; Papaevangelou, Vassiliki; Bianco, Veronique; Van der Wielen, Marie; Miller, Jacqueline M. (2011) [[30]](https://www.zotero.org/google-docs/?lGkX2h) |
|  | MenACWY + MenB | RCT (phase 3b) | 744 | 2.8 | 3.9 | Argentina, Mexico | Macias Parra, Mercedes; Gentile, Angela; Vazquez Narvaez, Jorge Alejandro; Capdevila, Alejandro; Minguez, Angel; Carrascal, Monica; Willemsen, Arnold; Bhusal, Chiranjiwi; Toneatto, Daniela (2018) [[31]](https://www.zotero.org/google-docs/?nhazoe) |
|  | MMR + PCV7 + Hib-MenCY | RCT | 280 | 12.0 | 13.0 | UK | Miller, Elizabeth; Andrews, Nick; Waight, Pauline; Findlow, Helen; Ashton, Lindsey; England, Anna; Stanford, Elaine; Matheson, Mary; Southern, Joanna; Sheasby, Elizabeth; Goldblatt, David; Borrow, Ray (2011) [[32]](https://www.zotero.org/google-docs/?ELjljT) |
|  | MenACWY + Tdap + MenB | RCT (phase 2) | 2648 | 120.0 | 143.0 | USA | Muse, Derek; Christensen, Shane; Bhuyan, Prakash; Absalon, Judith; Eiden, Joseph J.; Jones, Thomas R.; York, Laura J.; Jansen, Kathrin U.; O'Neill, Robert E.; Harris, Shannon L.; Perez, John L. (2016) [[33]](https://www.zotero.org/google-docs/?3WuJej) |
|  | MenACWY-TT + HepA-HepB | RCT (phase 3) | 611 | 132.0 | 204.0 | Denmark, Sweden | Ostergaard, Lars; Silfverdal, Sven-Arne; Berglund, Johan; Flodmark, Carl-Erik; West, Christina; Bianco, Veronique; Baine, Yaela; Miller, Jacqueline M. (2012) [[34]](https://www.zotero.org/google-docs/?PNc17x) |
|  | JE + Rabies | RCT (phase 2) | 200 | 12.0 | 18.0 | Thailand | Pengsaa, Krisana; Limkittikul, Kriengsak; Sabchareon, Arunee; Ariyasriwatana, Churdchoo; Chanthavanich, Pornthep; Attanath, Phanosri; Malerczyk, Claudius (2009) [[35]](https://www.zotero.org/google-docs/?0wFZs3) |
|  | MenACWY-TT + Tdap | RCT (phase 3) | 692 | 132.0 | 300.0 | Dominican Republic, Germany, Republic of Korea | Rivera, Luis; Schwarz, Tino F.; Kim, Kyung-Hyo; Kim, Yun-Kyung; Behre, Ulrich; Cha, Sung-Ho; Jo, Dae Sun; Lee, Jacob; Lee, Jin-Soo; Cheuvart, Brigitte; Jastorff, Archana; Van der Wielen, Marie (2018) [[36]](https://www.zotero.org/google-docs/?twMH5A) |
|  | DTaP-IPV/Hib + PVC7 + HepB | RCT (phase 4) | 376 | 2.0 | 2.0 | Canada | Scheifele, David W.; Halperin, Scott A.; Smith, Bruce; Ochnio, Jan; Meloff, Keith; Duarte-Monteiro, Denzyl (2006) [[37]](https://www.zotero.org/google-docs/?0S5P3Y) |
|  | HPV + HepB | RCT | 744 | 108.0 | 180.0 | Netherlands, Sweden | Schmeink, Channa E.; Bekkers, Ruud L. M.; Josefsson, Ann; Richardus, Jan H.; Berndtsson Blom, Katarina; David, Marie-Pierre; Dobbelaere, Kurt; Descamps, Dominique (2011 )[[38]](https://www.zotero.org/google-docs/?l1MyZ9) |
|  | DTaP-IPV/Hib + PCV7 | RCT | 231 | 2.0 | 15.0 | Germany | Schmitt, H. J.; Faber, J.; Lorenz, I.; Schmole-Thoma, B.; Ahlers, N. (2003) [[39]](https://www.zotero.org/google-docs/?EMWTNR) |
|  | 4vHPV + MenB | RCT (phase 2) | 2499 | 132.0 | 204.0 | USA | Senders, Shelly; Bhuyan, Prakash; Jiang, Qin; Absalon, Judith; Eiden, Joseph J.; Jones, Thomas R.; York, Laura J.; Jansen, Kathrin U.; O'Neill, Robert E.; Harris, Shannon L.; Ginis, John; Perez, John L. (2016) [[40]](https://www.zotero.org/google-docs/?jRFivr) |
|  | DTaP + MMRV + Hib-HepB | RCT | 1915 | 12.0 | 15.0 | USA | Shinefield, Henry; Black, Steve; Thear, Marci; Coury, Daniel; Reisinger, Keith; Rothstein, Edward; Xu, Jin; Hartzel, Jonathan; Evans, Barbara; Digilio, Laura; Schodel, Florian; Brown, Michelle L. Hoffman; Kuter, Barbara (2006) [[41]](https://www.zotero.org/google-docs/?WeiT0Z) |
|  | DTaP + Hib-HepB |  |  |  |  |  |  |
|  | MMR + VAR |  |  |  |  |  |  |
|  | MenACWY + DT/Td | RCT | 1754 | 42.0 | 215.0 | UK | Southern, Jo; Gelb, David; Andrews, Nick; Waight, Pauline A.; Morris, Rhonwen; Cartwright, Keith; Miller, Elizabeth (2006) [[42]](https://www.zotero.org/google-docs/?sQAlj6) |
|  | DTaP-HepB-IPV/Hib + HepA | RCT | 619 | 1.9 | 2.6 | Belgium, Germany | Stojanov, S.; Liese, J. G.; Belohradsky, B. H.; Vandermeulen, C.; Hoppenbrouwers, K.; Van der Wielen, M.; Van Damme, P.; Georges, B.; Dupuy, M.; Scemama, M.; Watson, M.; Fiquet, A.; Stek, J. E.; Golm, G. T.; Schodel, F. P.; Kuter, B. J. (2007) [[43]](https://www.zotero.org/google-docs/?z2VZby) |
|  | Tdap + IIV | Retrospective observational cohort | 36844 | 168.0 | 588.0 | USA | Sukumaran, Lakshmi; McCarthy, Natalie L.; Kharbanda, Elyse O.; Weintraub, Eric S.; Vazquez-Benitez, Gabriela; McNeil, Michael M.; Li, Rongxia; Klein, Nicola P.; Hambidge, Simon J.; Naleway, Allison L.; Lugg, Marlene M.; Jackson, Michael L.; King, Jennifer P.; DeStefano, Frank; Omer, Saad B.; Orenstein, Walter A. (2015) [[44]](https://www.zotero.org/google-docs/?Wx1lbq) |
|  | DTaP-HepB-IPV/Hib + PCV7 or PCV13 | Case reports | 883 | 0.0 | 24.0 | Italy | Trotta, Francesco; Santuccio, Carmella; Felicetti, Patrizia; Bella, Antonino; Rizzo, Caterina; Conti, Valentino; Monaco, Giuseppe; Russo, Francesca; Zanoni, Giovanna; Osbello, Loredana; Pascucci, Maria Grazia; Parrilli, Maria; Rossi, Marco (2015) [[45]](https://www.zotero.org/google-docs/?rLoPbW) |
|  | MenACWY + MMRV | RCT (phase 3) | 1000 | 12.0 | 23.0 | Finland | Vesikari, Timo; Karvonen, Aino; Bianco, Veronique; Van der Wielen, Marie; Miller, Jacqueline (2011) [[6]](https://www.zotero.org/google-docs/?DxjVA0) |
|  | MenACWY-TT + DTaP-HepB-IPV/Hib | RCT (phase 3) | 312 | 12.0 | 13.7 | Finland | Vesikari, Timo; Borrow, Ray; Da Costa, Xavier; Thomas, Stéphane; Eymin, Cécile; Boisnard, Florence; Lockhart, Stephen (2018) [[46]](https://www.zotero.org/google-docs/?XEEnAa) |
|  | MenACWY + Tdap | RCT (phase 4) | 1341 | 132.0 | 216.0 | USA | Weston, Wayde M.; Friedland, Leonard R.; Wu, Xiangfeng; Howe, Barbara (2011) [[47]](https://www.zotero.org/google-docs/?3A6MBx) |
|  | HPV + HepB | RCT | 1877 | 192.0 | 276.0 | Brazil, Czech Republic, Germany, USA | Wheeler, Cosette M.; Bautista, Oliver M.; Tomassini, Joanne E.; Nelson, Margaret; Sattler, Carlos A.; Barr, Eliav (2008) [[48]](https://www.zotero.org/google-docs/?tJgQvE) |
|  | Tdap + HPV | RCT (phase 3) | 1283 | 132.0 | 216.0 | USA | Wheeler, Cosette M.; Harvey, Bryan M.; Pichichero, Michael E.; Simon, Michael W.; Combs, Stephen P.; Blatter, Mark M.; Marshall, Gary S.; Catteau, Gregory; Dobbelaere, Kurt; Descamps, Dominique; Dubin, Gary; Schuind, Anne (2011) [[7]](https://www.zotero.org/google-docs/?Up1p0f) |
|  | DTaP-HepB-IPV/Hib + MMRV | RCT | 451 | 12.0 | 23.0 | Germany | Zepp, Fred; Behre, Ulrich; Kindler, Klaus; Laakmann, Karl-Heinz; Pankow-Culot, Heidemarie; Mannhardt-Laakmann, Wilma; Beckers, Francois; Descamps, Dominique; Willems, Paul (2007) [[49]](https://www.zotero.org/google-docs/?beorCT) |

*^#^ in co-administration as compared to separate administration*

** vaccinations*

**^+^** *age in months*

References

1. Reisinger, K.S.; Block, S.L.; Collins-Ogle, M.; Marchant, C.; Catlett, M.; Radley, D.; Sings, H.L.; Haupt, R.M.; Garner, E.I.O. Safety, tolerability, and immunogenicity of gardasil given concomitantly with menactra and adacel. *Pediatrics* **2010**, *125*, 1142–1151. doi:10.1542/peds.2009-2336.
2. Schilling, A.; Parra, M.M.; Gutierrez, M.; Restrepo, J.; Ucros, S.; Herrera, T.; Engel, E.; Huicho, L.; Shew, M.; Maansson, R.; et al. Coadministration of a 9-valent human papillomavirus vaccine with meningococcal and tdap vaccines. *Pediatrics* **2015**, *136*, e563–e572.
3. Cassidy, W.M.; Jones, G.; Williams, K.; Deforest, A.; Forghani, B.; Virella, G.; Venters, C. Safety and immunogenicity of concomitant versus nonconcomitant administration of hepatitis, B.; tetanus-diphtheria, and measles-mumps-rubella vaccines in healthy eleven- to twelve-year-olds. *J. Adolesc. Health* **2005**, *36*, 187–192. doi:10.1016/j.jadohealth.2004.02.021.
4. Olivier, B., Stojanov, B., Petersen, L. Immunogenicity, reactogenicity, and safety of a seven-valent pneumococcal conjugate vaccine (PCV7) concurrently administered with a fully liquid DTPa-IPV-HBV-Hib combination vaccine in healthy infants. *Vaccine* **2008**, *26*, 3142–3152.
5. Stockwell, M.S.; Broder, K.; LaRussa, P.; Lewis, P.; Fernandez, N.; Sharma, D.; Barrett, A.; Sosa, J.; Vellozzi, C. Risk of fever after pediatric trivalent inactivated influenza vaccine. *JAMA Pediatrics* **2014**, *168*, 211–219. doi:10.1001/jamapediatrics.2013.4469.
6. Vesikari, T.; Karvonen, A.; Borrow, R.; Kitchin, N.; Baudin, M.; Thomas, S.; Fiquet, A. Results from a randomized clinical trial of coadministration of RotaTeq, a pentavalent rotavirus vaccine, and NeisVac-C, a meningococcal serogroup C conjugate vaccine. *Clin. Vaccine Immunol.* **2011**, *18*, 878–884. doi:10.1128/CVI.00437-10.
7. Wheeler, C.M.; Harvey, B.M.; Pichichero, M.E.; Simon, M.W.; Combs, S.P.; Blatter, M.M.; Marshall, G.S.; Catteau, G.; Dobbelaere, K.; Descamps, D.; et al. Immunogenicity and safety of human papillomavirus-16/18 AS04-adjuvanted vaccine coadministered with tetanus toxoid, reduced diphtheria toxoid, and acellular pertussis vaccine and/or meningococcal conjugate vaccine to healthy girls 11 to 18 years of age: Results from a randomized open trial. *Pediatrics* *Infect. Dis. J.* **2011**, *30*, e225–e234. doi:10.1097/INF.0b013e31822d28df.
8. Shneyer, E.; Strulov, A.; Rosenfeld, Y. Reduced rate of side effects associated with separate administration of MMR and DTaP-Hib-IPV vaccinations. *Isr. Med. Assoc. J. IMAJ* **2009**, *11*, 735–738.
9. Tejedor, J.C.; Omenaca, F.; Garcia-Sicilia, J.; Verdaguer, J.; Van Esso, D.; Esporrin, C.; Molina, V. ; Muro, M.; Mares, J.; Enrubia, M.; et al. Immunogenicity and reactogenicity of a three-dose primary vaccination course with a combined diphtheria-tetanus-acellular pertussis-hepatitis B-inactivated polio-Haemophilus influenzae type b vaccine coadministered with a meningococcal C conjugate vaccine. *Pediatric Infect. Dis. J.* **2004**, *23*, 1109–1115.
10. Breiman, R.F.; Brooks, W.A.; Goswami, D.; Lagos, R.; Borja-Tabora, C.; Lanata, C.F.; Londono, J.A.C.; Lum, L.C.S.; Rappaport, R.; Razmpour, A.; et al. A multinational, randomized, placebo-controlled trial to assess the immunogenicity, safety, and tolerability of live attenuated influenza vaccine coadministered with oral poliovirus vaccine in healthy young children. *Vaccine* **2009**, *27*, 5472–5479. doi:10.1016/j.vaccine.2009.07.002.
11. Hesley, T.M.; Reisinger, K.S.; Sullivan, B.J.; Jensen, E.H.; Stasiorowski, S.; Meechan, C.; Chan, C.Y.; West, D.J. Concomitant administration of a bivalent Haemophilus influenzae type b-hepatitis B vaccine, measles-mumps-rubella vaccine and varicella vaccine: Safety, tolerability and immunogenicity. *Pediatric Infect. Dis. J.* **2004**, *23*, 240–245.
12. Leonardi, M.; Bromberg, K.; Baxter, R.; Gardner, J.L.; Klopfer, S.; Nicholson, O.; Brockley, M.; Trammel, J.; Leamy, V.; Williams, W.; et al. Immunogenicity and safety of MMRV and PCV-7 administered concomitantly in healthy children. *Pediatrics* **2011**, *128*, e1387–e1394. doi:10.1542/peds.2010-2132.
13. Tanaka, Y.; Yokokawa, R.; Rong, H.S.; Kishino, H.; Stek, J.E.; Nelson, M.; Lawrence, J. Concomitant administration of diphtheria, tetanus, acellular pertussis and inactivated poliovirus vaccine derived from Sabin strains (DTaP-sIPV) with pentavalent rotavirus vaccine in Japanese infants. *Hum. Vaccines Immunother.* **2017**, *13*, 1–7. doi:10.1080/21645515.2017.1279769.
14. Arguedas, A.; Soley, C.; Loaiza, C.; Rincon, G.; Guevara, S.; Perez, A.; Porras, W.; Alvarado, O.; Aguilar, L.; Abdelnour, A.; et al. Safety and immunogenicity of one dose of MenACWY-CRM, an investigational quadrivalent meningococcal glycoconjugate vaccine, when administered to adolescents concomitantly or sequentially with Tdap and HPV vaccines. *Vaccine* **2010**, *28*, 3171–3179. doi:10.1016/j.vaccine.2010.02.045.

15.

1. Ciarlet, M.; Sani-Grosso, R.; Yuan, G.; Liu, G.F.; Heaton, P.M.; Gottesdiener, K.M.; Arredondo, J.L.; Schodel, F. Concomitant use of the oral pentavalent human-bovine reassortant rotavirus vaccine and oral poliovirus vaccine. *Pediatric Infect. Dis. J.* **2008**, *27*, 874–880. doi:10.1097/INF.0b013e3181782780.
2. Clarke, E.; Saidu, Y.; Adetifa, J.U.; Adigweme, I.; Hydara, M.B.; Bashorun, A.O.; Moneke-Anyanwoke, N.; Umesi, A.; Roberts, E.; Cham, P.M.; et al. Safety and immunogenicity of inactivated poliovirus vaccine when given with measles-rubella combined vaccine and yellow fever vaccine and when given via different administration routes: A phase 4, randomised, non-inferiority trial in The Gambia. *Lancet Glob. Health* **2016**, *4*, e534–e547.
3. Cutland, C.L.; Nolan, T.; Halperin, S.A.; Kurugol, Z.; Ahmed, K.; Perrett, K.P.; Richmond, P.; Marshall, H.S:; Ceyhan, M.; Kolhe, D.; et al. Immunogenicity and safety of one or two doses of the quadrivalent meningococcal vaccine MenACWY-TT given alone or with the 13-valent pneumococcal conjugate vaccine in toddlers: A phase III, open-label, randomised study. *Vaccine* **2018**, *36*, 1908–1916. doi:10.1016/j.vaccine.2018.02.013.
4. Deichmann, K.A.; Ferrera, G.; Tran, C.; Thomas, S.; Eymin, C.; Baudin, M. Immunogenicity and safety of a combined measles, mumps, rubella and varicella live vaccine (ProQuad (R)) administered concomitantly with a booster dose of a hexavalent vaccine in 12-23-month-old infants. *Vaccine* **2015**, *33*, 2379–2386. doi:10.1016/j.vaccine.2015.02.070.
5. Durando, P.; Esposito, S.; Bona, G.; Cuccia, M.; Desole, M.G.; Ferrera, G.; Gabutti, G.; Pellegrino, A.; Salvini, F.; Henry, O.; et al. The immunogenicity and safety of a tetravalent measles-mumps-rubella-varicella vaccine when co-administered with conjugated meningococcal C vaccine to healthy children: A phase IIIb, randomized, multi-center study in Italy. *Vaccine* **2016**, *34*, 4278–4284. doi:10.1016/j.vaccine.2016.07.009.
6. Esposito, S.; Meregalli, E.; Daleno, C.; Ghio, L.; Tagliabue, C.; Valzano, A.; Serra, D.; Galeone, C.; Edefonti, A.; Principi, N. An open-label, randomized clinical trial assessing immunogenicity, safety and tolerability of pandemic influenza A/H1N1 MF59-adjuvanted vaccine administered sequentially or simultaneously with seasonal virosomal-adjuvanted influenza vaccine to paediatric kidney transplant recipients. *Nephrol. Dial. Transplant.* **2011**, *26*, 2018–2024. doi:10.1093/ndt/gfq657.
7. Esposito, S.; Tagliaferri, L.; Daleno, C.; Valzano, A.; Picciolli, I.; Tel, F.; Prunotto, G.; Serra, D.; Galeone, C.; Plebani, A.; et al. Pandemic influenza A/H1N1 vaccine administered sequentially or simultaneously with seasonal influenza vaccine to HIV-infected children and adolescents. *Vaccine* **2011**, *29*, 1677–1682. doi:10.1016/j.vaccine.2010.12.047.
8. Gasparini, R.; Conversano, M.; Bona, G.; Gabutti, G.; Anemona, A.; Dull, P.M.; Ceddia, F. Randomized trial on the safety, tolerability, and immunogenicity of MenACWY-CRM, an investigational quadrivalent meningococcal glycoconjugate vaccine, administered concomitantly with a combined tetanus, reduced diphtheria, and acellular pertussis vaccine in adolescents and young adults. *Clin. Vaccine Immunol. CVI* **2010**, *17*, 537–544. doi:10.1128/CVI.00436-09.
9. Gatchalian, S.; Leboulleux, D.; Desauziers, E.; Bermal, N.; Borja-Tabora, C. Immunogenicity and safety of a varicella vaccine, Okavax, and a trivalent measles, mumps and rubella vaccine, MMR-II, administered concomitantly in healthy Filipino children aged 12–24 months. *Southeast Asian J. Trop. Med. Public Health* **2003**, *34*, 589–597.
10. Gatchalian, S.; Tabora, C.; Bermal, N.; Leboulleux, D.; Desauziers, E. Immunogenicity and safety of a varicella vaccine (Okavax) and a trivalent measles, mumps, and rubella vaccine (Trimovax) administered concomitantly in healthy Filipino children 12-24 months old. *Am. J. Trop. Med. Hyg.* **2004**, *70*, 273–277.
11. Gatchalian, S.; Yao, Y.; Zhou, B.; Zhang, L.; Yoksan, S.; Kelly, K.; Neuzil, K.M.; Yaich, M.; Jacobson, J. Comparison of the immunogenicity and safety of measles vaccine administered alone or with live, attenuated Japanese encephalitis SA 14-14-2 vaccine in Philippine infants. *Vaccine* **2008**, *26*, 2234–2241. doi:10.1016/j.vaccine.2008.02.042.
12. Halperin, S.A.; Tapiero, B.; Dionne, M.; Meekison, W.; Diaz-Mitoma, F.; Zickler, P.; Rubin, E.; Embree, J.; Bhuyan, P.; Lee, A.; et al. Safety and immunogenicity of a toddler dose following an infant series of a hexavalent diphtheria, tetanus, acellular pertussis, inactivated poliovirus, Haemophilus influenzae type b, hepatitis B vaccine administered concurrently or at separate visits with a heptavalent pneumococcal conjugate vaccine. *Pediatric Infect. Dis. J.* **2014**, *33*, 73–80. doi:10.1097/01.inf.0000437806.76221.20.
13. Huang, L.-M.; Lin, T.-Y.; Chiu, C.-H.; Chiu, N.-C.; Chen, P.-Y.; Yeh, S.-J.; Boaz, M.; Hutagalung, Y.; Bouckenooghe, A.; Feroldi, E. Concomitant administration of live attenuated Japanese encephalitis chimeric virus vaccine (JE-CV) and measles, mumps, rubella (MMR) vaccine: Randomized study in toddlers in Taiwan. *Vaccine* **2014**, *32*, 5363–5369.
14. Jackson, L.A.; Yu, O.; Nelson, J.; Belongia, E.A.; Hambidge, S.J.; Baxter, R.; Naleway, A.; Nordin, J.; Baggs, J.; Iskander, J. Risk of medically attended local reactions following diphtheria toxoid containing vaccines in adolescents and young adults: A Vaccine Safety Datalink study. *Vaccine* **2009**, *27*, 4912–4916. doi:10.1016/j.vaccine.2009.06.038.
15. Kiely, M.; Billard, M.-N.; Toth, E.; Zafack, J.G.; Landry, M.; Skowronski, D.M.; De Serres, G. Investigation of an increase in large local reactions following vaccine schedule change to include DTaP-HB-IPV-Hib (Infanrix-hexa) and MMRV (ProQuad) at 18months of age. *Vaccine* **2018**, *36*, 6688–6694.
16. Knuf, M.; Pantazi-Chatzikonstantinou, A.; Pfletschinger, U.; Tichmann-Schumann, I.; Maurer, H.; Maurer, L.; Fischbach, T.; Zinke, H.; Pankow-Culot, H.; Papaevangelou, V.; et al. An investigational tetravalent meningococcal serogroups, A.; C, W-135 and Y-tetanus toxoid conjugate vaccine co-administered with Infanrix hexa is immunogenic, with an acceptable safety profile in 12-23-month-old children. *Vaccine* **2011**, *29*, 4264–4273. doi:10.1016/j.vaccine.2011.03.009.
17. Macias Parra, M.; Gentile, A.; Vazquez Narvaez, J.A.; Capdevila, A.; Minguez, A.; Carrascal, M.; Willemsen, A.; Bhusal, C.; Toneatto, D. Immunogenicity and safety of the 4CMenB and MenACWY-CRM meningococcal vaccines administered concomitantly in infants: A phase 3b, randomized controlled trial. *Vaccine* **2018**, *36*, 7609–7617.
18. Miller, E.; Andrews, N.; Waight, P.; Findlow, H.; Ashton, L.; England, A.; Stanford, E.; Matheson, M.; Southern, J.; Sheasby, E.; et al. Safety and immunogenicity of coadministering a combined meningococcal serogroup C and Haemophilus influenzae type b conjugate vaccine with 7-valent pneumococcal conjugate vaccine and measles, mumps, and rubella vaccine at 12 months of age. *Clin. Vaccine Immunol. CVI* **2011**, *18*, 367–372. doi:10.1128/CVI.00516-10.
19. Muse, D.; Christensen, S.; Bhuyan, P.; Absalon, J.; Eiden, J.J.; Jones, T.R.; York, L.J.; Jansen, K.U.; O'Neill, R.E.; Harris, S.L.; et al. A Phase 2, Randomized, Active-controlled, Observer-blinded Study to Assess the Immunogenicity, Tolerability and Safety of Bivalent rLP2086, a Meningococcal Serogroup B Vaccine, Coadministered With Tetanus, Diphtheria and Acellular Pertussis Vaccine and Serogroup, A.; C, Y and W-135 Meningococcal Conjugate Vaccine in Healthy US Adolescents. *Pediatric Infect. Dis. J.* **2016**, *35*, 673–682. doi:10.1097/INF.0000000000001124.
20. Ostergaard, L.; Silfverdal, S.-A.; Berglund, J.; Flodmark, C.-E.; West, C.; Bianco, V.; Baine, Y.; Miller, J.M. A tetravalent meningococcal serogroups, A.; C, W-135, and Y tetanus toxoid conjugate vaccine is immunogenic and well-tolerated when co-administered with Twinrix((R)) in subjects aged 11-17 years: An open, randomised, controlled trial. Vaccine 2012, 30, 774–783. doi:10.1016/j.vaccine.2011.11.051.
21. Pengsaa, K.; Limkittikul, K.; Sabchareon, A.; Ariyasriwatana, C.; Chanthavanich, P.; Attanath, P.; Malerczyk, C. A three-year clinical study on immunogenicity, safety, and booster response of purified chick embryo cell rabies vaccine administered intramuscularly or intradermally to 12- to 18-month-old Thai children, concomitantly with Japanese encephalitis vaccine. Pediatric Infect. Dis. J. 2009, 28, 335–337. doi:10.1097/INF.0b013e3181906351.
22. Rivera, L.; Schwarz, T.F.; Kim, K.-H.; Kim, Y.-K.; Behre, U.; Cha, S.-H.; Jo, D.S.; Lee, J.; Lee, J.-S.; Cheuvart, B.; et al. Immunogenicity and safety of the quadrivalent meningococcal vaccine MenACWY-TT co-administered with a combined diphtheria-tetanus-acellular pertussis vaccine versus their separate administration in adolescents and young adults: A phase III, randomized study. *Vaccine* **2018**, *36*, 4750–4758. doi:10.1016/j.vaccine.2018.04.034.
23. Scheifele, D.W.; Halperin, S.A.; Smith, B.; Ochnio, J.; Meloff, K.; Duarte-Monteiro, D. Assessment of the compatibility of co-administered 7-valent pneumococcal conjugate, DTaP.IPV/PRP-T Hib and hepatitis B vaccines in infants 2-7 months of age. *Vaccine* **2006**, *24*, 2057–2064. doi:10.1016/j.vaccine.2005.11.021.
24. Schmeink, C.E.; Bekkers, R.L.M.; Josefsson, A.; Richardus, J.H.; Berndtsson Blom, K.; David, M.-P.; Dobbelaere, K.; Descamps, D. Co-administration of human papillomavirus-16/18 AS04-adjuvanted vaccine with hepatitis B vaccine: Randomized study in healthy girls. *Vaccine* **2011**, *29*, 9276–9283. doi:10.1016/j.vaccine.2011.08.037.
25. Schmitt, H.J.; Faber, J.; Lorenz, I.; Schmole-Thoma, B.; Ahlers, N. The safety, reactogenicity and immunogenicity of a 7-valent pneumococcal conjugate vaccine (7VPnC) concurrently administered with a combination DTaP-IPV-Hib vaccine. *Vaccine* **2003**, *21*, 3653–3662.
26. Senders, S.; Bhuyan, P.; Jiang, Q.; Absalon, J.; Eiden, J.J.; Jones, T.R.; York, L.J.; Jansen, K.U.; O'Neill, R.E.; Harris, S.L.; et al. Immunogenicity, Tolerability and Safety in Adolescents of Bivalent rLP2086, a Meningococcal Serogroup B Vaccine, Coadministered with Quadrivalent Human Papilloma Virus Vaccine. *Pediatric Infect. Dis. J.* **2016**, *35*, 548–554. doi:10.1097/INF.0000000000001072.
27. Shinefield, H.; Black, S.; Thear, M.; Coury, D.; Reisinger, K.; Rothstein, E.; Xu, J.; Hartzel, J.; Evans, B.; Digilio, L.; et al. Safety and immunogenicity of a measles, mumps, rubella and varicella vaccine given with combined Haemophilus influenzae type b conjugate/hepatitis B vaccines and combined diphtheria-tetanus-acellular pertussis vaccines. Pediatric Infect. Dis. J. 2006, 25, 287–292. doi:10.1097/01.inf.0000207857.10947.1f.
28. Southern, J.; Gelb, D.; Andrews, N.; Waight, P.A.; Morris, R.; Cartwright, K.; Miller, E. Reactogenicity of meningococcal C conjugate vaccines when administered at the same time as, a month prior to or after, tetanus and diphtheria booster vaccinations. Hum. Vaccin 2006, 2, 237–242.
29. Stojanov, S.; Liese, J.G.; Belohradsky, B.H.; Vandermeulen, C.; Hoppenbrouwers, K.; Van der Wielen, M.; Van Damme, P.; Georges, B.; Dupuy, M.; Scemama, M.; et al. Administration of hepatitis A vaccine at 6 and 12 months of age concomitantly with hexavalent (DTaP-IPV-PRP approximately T-HBs) combination vaccine. Vaccine 2007, 25, 7549–7558. doi:10.1016/j.vaccine.2007.08.028.
30. Sukumaran, L.; McCarthy, N.L.; Kharbanda, E.O.; Weintraub, E.S.; Vazquez-Benitez, G.; McNeil, M.M.; Li, R.; Klein, N.P.; Hambidge, S.J.; Naleway, A.L.; et al. Safety of Tetanus Toxoid, Reduced Diphtheria Toxoid, and Acellular Pertussis and Influenza Vaccinations in Pregnancy. Obstet Gynecol. 2015, 126, 1069–1074. doi:10.1097/AOG.0000000000001066.
31. Trotta, F.; Santuccio, C.; Felicetti, P.; Bella, A.; Rizzo, C.; Conti, V.; Monaco, G. ; Russo, F.; Zanoni, G.; Osbello, L.; et al. Comparative safety evaluation of 7-valent and 13-valent pneumococcal vaccines in routine paediatric vaccinations in four Italian regions, 2009 to 2011. Eurosurveillance 2015, 20, 21041.
32. Vesikari, T.; Borrow, R.; Da Costa, X.; Thomas, S.; Eymin, C.; Boisnard, F.; Lockhart, S. Concomitant administration of a fully liquid ready-to-use DTaP-IPV-HB-PRP-T hexavalent vaccine with a meningococcal ACWY conjugate vaccine in toddlers. *Vaccine* **2018**, *36*, 8019–8027.
33. Weston, W.M.; Friedland, L.R.; Wu, X.; Howe, B. Immunogenicity and reactogenicity of co-administered tetanus-diphtheria-acellular pertussis (Tdap) and tetravalent meningococcal conjugate (MCV4) vaccines compared to their separate administration. *Vaccine* **2011**, *29*, 1017–1022. doi:10.1016/j.vaccine.2010.11.057.
34. Wheeler, C.M.; Bautista, O.M.; Tomassini, J.E.; Nelson, M.; Sattler, C.A.; Barr, E. Safety and immunogenicity of co-administered quadrivalent human papillomavirus (HPV)-6/11/16/18 L1 virus-like particle (VLP) and hepatitis B (HBV) vaccines. *Vaccine* **2008**, *26*, 686–696. doi:10.1016/j.vaccine.2007.11.043.
35. Zepp, F.; Behre, U.; Kindler, K.; Laakmann, K.-H.; Pankow-Culot, H.; Mannhardt-Laakmann, W.; Beckers, F.; Descamps, D.; Willems, P. Immunogenicity and safety of a tetravalent measles-mumps-rubella-varicella vaccine co-administered with a booster dose of a combined diphtheria-tetanus-acellular pertussis-hepatitis B-inactivated poliovirus-Haemophilus influenzae type b conjugate vaccine in healthy children aged 12-23 months. *Eur. J. Pediatrics* **2007**, *166*, 857–864. doi:10.1007/s00431-007-0506-z.

| 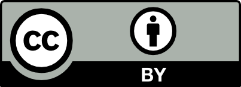 | © 2018 by the authors. Submitted for possible open access publication under the terms and conditions of the Creative Commons Attribution (CC BY) license (http://creativecommons.org/licenses/by/4.0/). |
| --- | --- |
